# Functional Properties of Foods and Beverages

**DOI:** 10.3390/foods13172763

**Published:** 2024-08-30

**Authors:** Arshad Mehmood Abbasi, Xinbo Guo, Yongsheng Chen

**Affiliations:** 1 Department of Environmental Sciences, COMSATS University Islamabad, Abbottabad Campus, Abbottabad 22060, Pakistan; 2 School of Food Science and Engineering, South China University of Technology, Guangzhou 510006, China; guoxinbo@scut.edu.cn; 3 Department of Food Science and Engineering, Jinan University, Guangzhou 510632, China; chysh11@126.com

## 1. Introduction

The conception “*Let food be thy medicine and medicine be thy food*” introduced by the father of medicine “Hippocrates” highlights the significance of bioactive substances in human health. Nutraceuticals including functional foods, medicinal foods, and dietary supplements are the products developed from various plant- or animal-based food sources [1]. They contribute significantly to preventing diseases and promoting health. The functional properties and nutraceutical potential of plants are primarily attributed to phytonutrients and a wide array of bioactive compounds, often referred to as ProHealth nutrients. These naturally occurring, non-nutritive bioactive compounds (secondary metabolites/phytochemicals) are not essential for basic human survival but offer substantial health benefits [2].

Functional foods and beverages (FFsBs) encompass raw, cooked, or processed edible substances that provide health benefits beyond basic nutrition. Being rich in bioactive compounds, FFsBs contribute significantly to promoting well-being, preventing health disorders, and enhancing the body’s normal functioning [3]. Thousands of bioactive phytochemicals, including polyphenolics, carotenoids, vitamins, glucosinolates, probiotics, and prebiotics, are found in various fruits, vegetables, grains, seeds, teas, and wines. Due to their antioxidant, antimicrobial, anti-inflammatory, anti-aging, and anticancer properties, these bioactive substances prevent and reduce the risk of chronic health disorders such as cardiovascular diseases, neurodegenerative disorders, early aging, inflammation, Alzheimer’s disease, eye infections, and digestive disorders [4,5].

Incorporating functional foods and beverages into our diets represents a necessary progression in our approach to nutrition and health. Research on the functional properties of foods and beverages reveals the complex relationships between physiological activities and the bioactive potential of compounds. As the potential for functional foods and nutraceuticals to improve public health becomes increasingly evident, interest in this field continues to grow [6].

Nutraceuticals, functional foods, or medicinal foods represent a fascinating area for researchers working across various disciplines, including food science and technology, biochemistry, pharmaceutics, nutraceutics, pharmacology, natural product chemistry, and biotechnology. Moreover, industries such as food and beverages, pharmaceuticals, and nutraceuticals are increasingly focusing on research that explores the functional properties and nutraceutical potential of bio-resources.

## 2. Overview of the Contributions

In this Special Issue, a total of ten articles are collected, of which nine are original research articles and one is review article. All studies are mainly focused on the phytochemical composition and bioactive potential of foods and beverages.

A study conducted by Koh et al. [contribution 1] was focused on the development of Gamma-aminobutyric acid (GABA)-enriched dark chocolate and its application in regulating hypertension. The GABA-enriched dark chocolate was prepared by replacing sugar syrup with pure GABA powder at various concentrations. The addition of GABA provides substantially increased hardness, and the angiotensin-converting enzyme inhibitory effect of chocolate. Moreover, enhanced shelf-life test results showed that all the chocolates were microbiologically safe for consumption for at least 21 days.

Although different polyphenols like flavonoids are well recognized because of their physiological properties, different physiochemical properties, specifically surface activities, have rarely been explored. Therefore, Lu et al. [contribution 2] manufactured and characterized a natural quercetin self-stabilizing Pickering emulsion using an antisolvent precipitation method. Their findings showed that natural quercetin has a good capability to stabilize Pickering emulsion that may have potential application to develop novel and functional emulsion foods.

An investigation by Hussain et al. [contribution 3], was focused on the ethnomycological knowledge specifically in context of 34 edible mushrooms found in Swat region of Pakistan. *Agaricus bisporus*, *Auricularia* sp., *Flammulina velutipes*, *Ganoderma lucidum*, *Morchella angusticeps*, *M. esculenta*, *Pleurotus* sp. and *Sanghuangporus sanghuang* were the most cited edible and medicinal mushrooms. The authors suggested that the domestication and commercialization of wild edible mushrooms in this region could contribute significantly to the socio-economic development of the local communities.

One of the major causes of dental caries is oral biofilms (BFs) formed by *Streptococcus mutans.* To address this issue, Goto et al. [contribution 4] prepared a Roasted Green Tea (RGT) and examined its active compounds, and its inhibitory potential against *S. mutans* biofilms. They reported potent antibiofilm activity in RGT fractions having medium to high hydrophobicity and that are rich in phenolic hydroxyl groups. Their findings revealed RGT as a safe and effective anti-caries agent in future applications.

Lu et al. [contribution 5], synthesized a dihydromyricetin (DMY)/α-lactoalbumin (α-La) covalent complex by the alkaline method and evaluated its characterization and application in nano-emulsions. Their findings revealed strong associations between DMY and the total phenolic content of the complex. Furthermore, the DMY/α-La complex depicted spectacular antioxidant, free radical scavenging and glucosidase inhibitory activities. The authors suggested that the synthesized complex can be used as an emulsifier to stabilize the β-carotene-loaded nano-emulsion. Their findings could be valuable in developing a new food delivery system.

Qain et al. [contribution 6] systematically compared the physicochemical properties, volatile metabolites, and amino acids present in twenty types of Hakka *Huangjiu* from the Guangdong region of China. Among the studied types of Hakka *Huangjiu*, LPSH, ZJHL-1, and GDSY-1 had the lowest sugar content, whereas significant variations were observed in the organic acids, amino acids, and volatile profiles of targeted samples. The gas chromatography olfactometry revealed that most of the aroma-active ingredients were bestowed with a pleasant fruity aroma.

The sedimentation of solid particles in a lotus seed and lily bulb beverage (LLB) is a challenge. To address this issue, an LLB was homogenized at different pressures to examine the changes in the microstructure, distribution of particle size, rheologic behavior, sedimentation index, turbidity, physicochemical properties, and sensory quality of the beverage [Su et al., contribution 7]. The suspended particles and microstructures decomposed with increasing homogenization at high pressure. The LLB exhibited shear-thinning, weak gelation, and rheological properties. Specifically, at 60 MPa homogenization pressure, the lowest sedimentation rate and highest sensory estimation were observed in the sample.

The study conducted by Qiu et al. [contribution 8] demonstrated a significant increase in the concentration of bioactive compounds and antioxidant properties, while showing a considerable reduction in the microbial counts of blackcurrant juice after thermo-sonication and pasteurization treatments. It was suggested that thermo-sonication is more effective than pasteurization for improving the quality of the method of making blackcurrant juice.

Zhang et al. [contribution 9] compared the phytochemical composition and bioactive potential in the hydrophilic and hydrophobic extracts of four herbal teas (*Mallotus*, *Cyclocarya*, *Rubus*, and Vine) originating from China. This study reveals the positive impact of herbal tea intake on postprandial blood glucose regulation. Specifically, Vine depicted noticeable glucose-regulating effects in aqueous and ethanolic infusions. Although ethanolic extracts yielded maximum phytochemicals, remarkably, aqueous extracts exhibited potent antioxidant activities. The findings of this investigation stipulate that aqueous extracts of herbal teas are rich in natural antioxidants and bioactive phytochemicals beneficial for hyperglycemia individuals.

A fermented beverage “Kombucha” has its origin in China and has spread globally. Barros et al. [contribution 10] conducted a consolidative review on alternative substrates to improve fermented beverages analogous to Kombucha, specifically from plants available in the Amazonia biome. About 37 new substrates, of which ~29% are found in the Amazon, were highlighted. The authors suggested that to produce kombucha-analogous beverages, sucrose (carbon/energy source) infusions prepared at 90–100 °C to increase contents of phenolic compounds and 14-day fermentation at 25–28 °C were the typical conditions. This review also reported gaps in the literature including the deficiency of reliable data about chemical composition, sensory aspects, biological properties, and market strategies for fermented beverages analogous to kombucha produced with alternative substrates.
